# The Wnt Signalling Pathway: A Tailored Target in Cancer

**DOI:** 10.3390/ijms21207697

**Published:** 2020-10-18

**Authors:** Malvina Koni, Veronica Pinnarò, Maria Felice Brizzi

**Affiliations:** Department of Medical Sciences, University of Turin, Corso Dogliotti 14, 10126 Turin, Italy; malvina.koni@unito.it (M.K.); veronica.pinnaro@edu.unito.it (V.P.)

**Keywords:** Wnt/β-catenin dependent pathway, Wnt/β-catenin independent pathway, colorectal cancer, breast cancer, ovarian cancer, extracellular vesicles

## Abstract

Cancer is one of the greatest public health challenges. According to the World Health Organization (WHO), 9.6 million cancer deaths have been reported in 2018. The most common cancers include lung, breast, colorectal, prostate, skin (non-melanoma) and stomach cancer. The unbalance of physiological signalling pathways due to the acquisition of mutations in tumour cells is considered the most common cancer driver. The Wingless-related integration site (Wnt)/β-catenin pathway is crucial for tissue development and homeostasis in all animal species and its dysregulation is one of the most relevant events linked to cancer development and dissemination. The canonical and the non-canonical Wnt/β-catenin pathways are known to control both physiological and pathological processes, including cancer. Herein, the impact of the Wnt/β-catenin cascade in driving cancers from different origin has been examined. Finally, based on the impact of Extracellular Vesicles (EVs) on tumour growth, invasion and chemoresistance, and their role as tumour diagnostic and prognostic tools, an overview of the current knowledge linking EVs to the Wnt/β-catenin pathway is also discussed.

## 1. Introduction

The human wingless-related integration site (Wnt) genes encode 19 evolutionarily conserved glycoproteins with 22-24 Cys residues. In the endoplasmic reticulum (ER), the Wnt ligands are post-translationally acetylated by porcupine, a membrane associated O-acyl transferase. Acetylation leads to palmitoylation, which is required for the release and binding of Wnt to the frizzled (*FZD*) receptors. This, in turn, drives the biological response [1].

The Wnt signalling pathway regulates crucial cellular processes including cell fate determination, organogenesis during embryonic development, normal adult homeostasis, motility, polarity and stem cell renewal [2]. Moreover, its contribution in cancer has been extensively investigated [3].

The Wnt pathway has been widely studied and reviewed, and a general understanding of the transduction cascade has been clarified. The Wnt cascade has been subdivided into different branches due to its complexity [4,5]. They include the canonical Wnt/β-catenin (Wnt/β-catenin dependent pathway) and the non-canonical Wnt/β-catenin pathway (β-catenin-independent pathway). The latter was further allocated into two additional branches, the planar cell polarity (PCP) and the Wnt/calcium pathways [2]. Both of them contribute to cancer development and dissemination.

The aim of the present review is to provide an overview of the current knowledge about the Wnt signalling pathway in tumour development and progression. Tumours from different origin are discussed. Although the canonical and the non-canonical Wnt/β-catenin pathway work together to control physiological and pathological processes [2], data related to each one are independently debated. Finally, the contribution of extracellular vesicles (EVs) in triggering the Wnt/β-catenin cascade is also analyzed.

## 2. Wnt Canonical Pathway: β-Catenin Dependent

**Figure 1:**
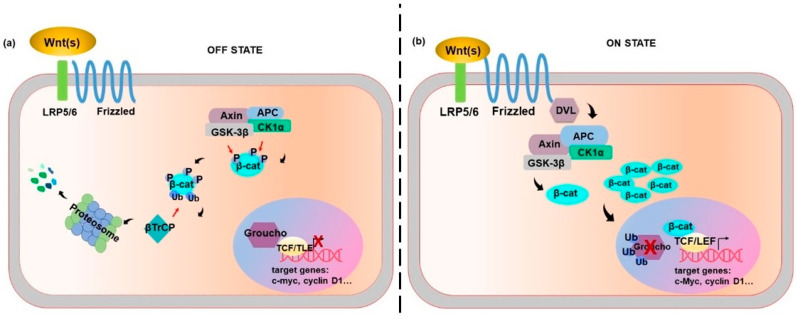
The Canonical Wnt signalling pathway. (**a**) OFF STATE. In the absence of Wnt ligands, β-catenin moves to the “destruction complex” consisting of casein kinase 1α (*CK1α*), glycogen synthase kinase 3 β (*GSK-3β*) and two scaffolds: axis Inhibition (*Axin*), and adenomatous polyposis coli (*APC*). β-catenin undergoes phosphorylation at Ser45 residue by *CK1α* and at Ser33, Ser37 and Thr41 residues by *GSK-3β*. Then, the E3 ubiquitin ligase β-transducin repeat-containing protein (*βTrCP*) marks β-catenin ubiquitination and proteasomal degradation. This prevents β-catenin nuclear accumulation while allowing chromatin compaction and Groucho-mediated promoter repression. (**b**) ON STATE. The Wnt ligands bind to frizzled (*FZD*) receptor and the low-density-lipoprotein-related protein 5/6 (*LRP5/LRP6*); this results in dishevelled (*DVL*) phosphorylation and β-catenin release from the “destruction complex”, allowing β-catenin accumulation and nuclear translocation. In the nucleus, the Groucho repressor undergoes displacement, allowing β-catenin to interact with T-cell factor/lymphoid enhancer factor (*TCF/LEF*), chromatin remodeling and transcription of genes such as *c-myc* and *cyclin D1*.

The canonical pathway turns around the β-catenin intracellular level (Figure 1). In the absence of Wnt proteins, the β-catenin “destruction complex” keeps low β-catenin in the cell. The “destruction complex” mainly consists of two kinases: casein kinase 1α (*CK1α*), glycogen synthase kinase 3 β (*GSK-3β*) and two scaffolds: axis inhibition (*Axin*), and adenomatous polyposis coli (*APC*). Firstly, β-catenin undergoes phosphorylation by *CK1α* at serine 45 (Ser45), Ser33, Ser37 and threonine 41 (Thr41) by *GSK-3β*. Then, the E3 ubiquitin ligase, denoted as β-transducin repeat-containing protein (*βTrCP*), marks β-catenin ubiquitination and degradation [1]. This prevents β-catenin nuclear translocation while allowing histone deacetylation and chromatin compaction by the Groucho repressor, translating into the inhibition of gene transcription [6] (Figure 1a).

The activation of the canonical Wnt signal requires both the *FZD* family receptors and the low-density-lipoprotein-related protein 5/6 (*LRP5/LRP6*) co-receptors, phosphorylation of which is essential for receptor activation. Wnt binding to its receptor results in dishevelled (*DVL*) phosphorylation, leading to *Axin* de-phosphorylation and decline of its cytoplasmic content [7]. Thereby, β-catenin can be released from the “destruction complex”, and its degradation prevented while stabilization is allowed. Accumulation of β-catenin turns into its nuclear translocation [7].

Although several nuclear β-catenin binding partners have been involved in the control of gene transcription, the most relevant β-catenin partners are the members of the T-cell factor/lymphoid enhancer factor (*TCF/LEF*) family of transcription factors [7]. This complex binds to the promoter region of target genes and regulates their transcription. 

Once in the nucleus, the engagement of β-catenin transiently converts the *TCF/LEF* into transcriptional activators, which displace Groucho and induce chromatin remodelling and transcriptional activity (Figure 1b).

A number of genes are targeted by Wnt-β-catenin. Among them, genes involved in positive- and negative-feedback regulation, cell-cycle progression, and stem cell homeostasis are the most commonly included genes.

## 3. Wnt Non-Canonical Pathways: Wnt/Planar Cell Polarity (PCP) and Wnt/Calcium 

**Figure 2:**
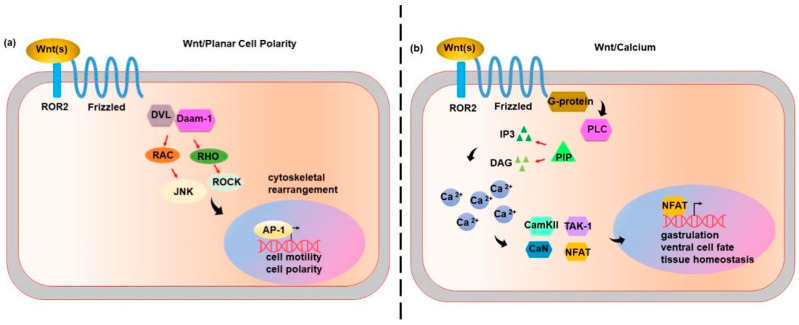
The Wnt non-canonical signalling pathways. (**a**) Wnt/planar cell polarity (PCP) pathway. Wnt ligands bind to *FZD* receptors and co-receptor RAR-related orphan receptor (*ROR*) and convey the signal to *DVL*. *DVL* forms the Disheveled associated activator of morphogenesis 1 (*DVL-Daam-1*) complex, which triggers *RhoA*, *RHO* and *ROCK* to control cytoskeletal rearrangement. On the other hand, *DVL* triggers *RAC*, *JNK* and *AP-1* involved in cell motility and polarity. (**b**) Wnt/Calcium pathway. Wnt ligands bind to *FZD* and activate the phospholipase C (*PLC*), which hydrolyses the phosphatidylinositol (4,5)-biphosphates (*PIP2*) to inositol (1,4,5)-triphosphates (*IP3*) and diacylglycerol (*DAG*). This translates into intracellular calcium release and the activation of *CaN* and *CamKII*. The calmodulin activation stimulates *TAK-1* and *NLK* activity. *CaN* activates the *NFAT*, which moves to the nucleus and modulates the expression of genes involved in the control of gastrulation, ventral cell fate and tissue homeostasis.

To date, the canonical Wnt/β-catenin pathway is much better characterized than the non-canonical one (Figure 2). 

In the non-canonical PCP pathway, Wnt ligands bind to *FZD* receptors and co-receptor protein tyrosine kinase 7 (*PTK7*), RAR-related orphan receptor (*ROR*) or the receptor like tyrosine kinase (*RYK*) and convey the signal to DVL. On the one side, *DVL* forms the disheveled associated activator of morphogenesis 1 (*DVL-Daam-1*) complex, which triggers a small guanosine-5’-triphosphate (GTP) GTPase, such as ras homolog gene family member A (*RhoA*), RHO and RHO-associated kinase (*ROCK*). *DVL* also triggers ras-related C3 botulinum toxin substrate (*RAC*), JUN-N-terminal kinase (*JNK*) and the activator protein-1 (*AP-1*). [7] The PCP pathway is involved in the cytoskeletal rearrangement, cell motility and co-ordinates cell polarity. In vertebrates, the PCP pathway is also required for morphology and migration of dorsal mesodermal cells undergoing gastrulation, hair follicle organization, and orientation of stereocilia in the sensory epithelium of the inner ear [8] (Figure 2a).

In the calcium-dependent pathway, Wnt ligands bind to *FZD* and activate the phospholipase C (*PLC*), which hydrolyses the phosphatidylinositol (4,5)-biphosphates (*PIP2*) to inositol (1,4,5)-triphosphates (*IP3*) and diacylglycerol (*DAG*). This translates into the release of the intracellular calcium and the activation of both calcineurin (*CaN*) and calcium/calmodulin-dependent kinase II (*CamKII*). Moreover, the activation of calmodulin promotes the activation of the TGF-β-Activated kinase 1 (*TAK-1*) and nemo-like kinase (*NLK*), thereby antagonizing and neutralizing the canonical Wnt/β-catenin cascade. *CaN* activates the nuclear factor of activated T-cells (*NFAT*), which moves to the nucleus and regulates the expression of target genes [7] (Figure 2b). The calcium-dependent pathway plays a crucial role in several processes, including early pattern formation during gastrulation [2], ventral cell fate [9], dorsal axis formation [10], and tissue homeostasis [11].

## 4. Colorectal Cancer

Colorectal cancer (CRC) is one of most common cancers worldwide and represents a deep cause of cancer mortality [12] with a rapid increase in incidence and death rate [13]. Dienstmann et al. [14] established a new classification of CRCs into four consensus molecular subtypes (*CMSs*). Among them *CMS2*, *CMS3*, and *CMS4* have a higher rate of *APC* mutations (over 50%) compared to *CMS1*. Each *CMS* has unique features: *CMS1* (MSI Immune, 14%): hyper- mutated, microsatellite instability, strong immune activation; *CMS2* (Canonical, 37%): epithelial, chromosomally unstable, marked Wnt and myc signalling activation; *CMS3* (Metabolic, 13%): epithelial, metabolic dysregulation; and *CMS4* (Mesenchymal, 23%): a prominent transforming growth factor β (*TGFβ*) activation, stromal invasion, and angiogenesis. Samples with combined features (13%) represent transition phenotypes or are supposed to reflect the intra-tumour heterogeneity [14].

The heterogeneous genetic ground underlying CRC initiation and progression mainly involves gene fusion, deletion or amplification, somatic gene mutations and epigenetic alterations. Wnt/β-catenin signalling has emerged as one of the most significant biological pathways in both the physiological setting and in CRC development. Almost all CRCs are characterized by a hyper-active Wnt/β-catenin pathway, which, in many cases, is considered the most critical cancer initiating and driving event. Proteins and miRNAs guiding the Wnt/β-catenin pathway and proposed as potential CRC therapeutic targets are discussed.

## 5. Canonical Wnt/β-Catenin Pathway and CRC

Ring finger protein 6 (*RNF6*) is an oncogene frequently upregulated by gene amplification in primary CRC. Moreover, *APC* mutation and *RNF6* copy number amplification were commonly found in CRC patients. *RNF6* is a RING-domain E3 ubiquitin ligase and exerts its pro-metastatic effects by promoting CRC cell growth, cell-cycle progression, and epithelial to mesenchymal transition (EMT). Furthermore, *RNF6* expression and its gene amplification have been considered independent patients‘ prognostic factors. *RNF6* mediates the polyubiquitination of the transducin-like enhancer of split 3 (*TLE3*), a transcriptional repressor of the β-catenin/*TCF4* complex, and its proteasome degradation. The lack of *TLE3/TCF4/LEF* interaction enhances the Wnt/β-catenin transcriptional activity, and the expression of its downstream target genes [15] (Table 1).

The leucine-rich repeat-containing G-protein coupled receptor 5 (*LGR5*) is a Wnt/β-catenin target gene implicated in cancer cell proliferation and migration. It has been reported that *LGR5* is highly expressed in CRC tissues compared to the healthy ones. A decline in β-catenin and *c-myc* mRNA expression were detected by knocking-down *LGR5* expression, suggesting that it may regulate the Wnt/β-catenin activity by modulating the expression of β-catenin. Furthermore, since targeting *LGR5* improves the response to chemotherapy, *LGR5* has been proposed as a novel therapeutic target in CRC [16] (Table 1).

The β-catenin and RAS signalling pathways are frequently associated with the development and progression of several different cancers. They mainly act on cancer stem cell (CSC) expansion. High levels of β-catenin and RAS proteins are considered the major drivers of CSC expansion and cancer dissemination and are associated with poor patient’s outcome [55]. 

Targeting the CSC pool without affecting the somatic stem cell (SSC) niche is one of the major goals of recent decades. As reported by Lenz et al. [56], the β-catenin antagonist molecule, ICG-001, effectively prevented the interplay between β-catenin and its coactivator cAMP response element binding protein (CREB)-binding protein (*CBP*). Moreover, ICG-001 effectively and without side effects abrogated drug-resistant cells. On the same line, PRI-724, a second generation of *CBP*/β-catenin antagonist, was found safe in pre-clinical studies and displayed an acceptable toxicity profile.

Yu et al. [18] investigated the traf2- and nck-interacting kinase (*TNIK*) amplification and its role in tumor progression by applying siRNA technology, while Masuda et al. [19] have generated a small molecule denoted as NCB-0846 acting as *TNIK* inhibitor. *TNIK* selectively binds both to *TCF4* and β-catenin in order to promote cancer cell growth via Wnt/β-catenin cascade and drives colorectal CSC expansion. The NCB-0846 inhibitor was effective in interfering with *TNIK* activity tumour growth. 

KYA1797K, a small molecule identified by Cha et al. [57], was found effective in suppressing CRC growth due to the activation of *GSK-3β* via Axin binding and β-catenin/RAS destabilization. In line with this observation, treatment with KYA1797K abrogated CRC stem cell features both in vitro and in vivo. Mechanistically, KYA1797K pushes β-catenin and RAS towards the *Axin* binding [20] (Table 1).

In the last decade, miRNAs have gained particular attention in cancer [58]. miRNA profiling has been linked to cancer types, stage, and invasion [59]. Moreover, oncogenic or tumour suppressive actions have been linked to miRNA expression. For these reasons, miRNAs are considered valuable tools for cancer diagnosis and prognosis and therefore useful therapeutic targets (Table 2). 

Sun and co-workers [21] identified miR-144-3p as a new biomarker for CRC diagnosis and response to treatment. miR-144-3p was found downregulated and associated with CRC pathological stages in CRC patients. Interestingly, miR-144-3p overexpression reduced CRC cell proliferation by delaying G1/S phase transition in tumour cells. On the contrary, the B-cell lymphoma 6 protein (*BCL6*), a nuclear protein belonging to the BTB/POZ/zinc finger (*ZF*) family of transcription factors, was found upregulated and surprisingly post-transcriptionally regulated by miR-144-3p. Previous studies revealed that *BCL6* is involved in the control of cell cycle progression and differentiation [22,23]. Indeed, miR-144-3p/*BCL6* co-operate to inhibit cellular proliferation, development, and progression of CRC by interfering with *c-myc* and *cyclin D1* expression [21] (Table 1).

miR-377-3p displays an ambiguous role in CRC. Liu and colleagues [60] uncovered that upregulation of miR-377-3p promotes G1-S phase transition, cell expansion and EMT, while repressing apoptosis in CRC patients. Moreover, *GSK-3β*, a direct miR-377-3p target, was found upregulated upon miR-377-3p overexpression. These data suggest that a complex regulatory network boosting tumour progression is associated with the expression of miR-377-3p in CRC. 

Conversely, in a recent study, Huang et al. [61] have shown that miR-377-3p, significantly reduced in CRC patients, is involved in the control of proliferation, migration and chemo resistance, particularly at advanced tumour stage. The authors investigated miR-377 functions and mechanism of action in CRC cells. The zinc finger E-box binding homeobox 2 (*ZEB2*) and the X-linked inhibitor of apoptosis protein (*XIAP*) are two positive regulators of the Wnt/β-catenin cascade [24,25]. In CRC, *ZEB2* enables tumour progression and invasion, whereas *XIAP* promotes cell proliferation and chemoresistance. De facto, miR-377-3p overexpression was found to suppress the malignant CRC phenotype, as well as cell proliferation, invasion and drug resistance by directly targeting the 3’ UTR sequence of both *ZEB2* and *XIAP* mRNAs. Since miR-377-3p/*ZEB2-XIAP* inhibited CRC progression by reducing Wnt/β-catenin-associated gene expression (e.i. *cyclin D1*, *Axin2*, *TCF1*, *SOX2*, c-*myc*, matrix metalloproteinase-2 (*MMP-2*), *MMP-9*, CD44, vascular endothelial growth factor (*VEGF*), and Twist), approaches involving increasing its expression have been proposed for novel therapeutic options (Table 1).

Functional experiments showed that miR-520e plays a pivotal role in regulating CRC cell proliferation, colony formation and invasion [62]. Moreover, it has been reported that low miR-520e expression is associated with the increased CRC growth and migration. The astrocyte elevated gene-1 (*AEG-1*), which acts as an oncogene [63], is a direct miR-520e target in CRC. Cells overexpressing miR-520e displayed lower *GSK-3β* phosphorylation and β-catenin expression. Mechanistically, it was found that miR-520e regulates cancer cell behaviour by targeting *AEG-1*, which in turn inactivates the Wnt/β-catenin signalling and the transcription of its downstream genes. Hence, miR-520e overexpression could represent a promising therapeutic target in CRC by *AEG-1* suppression.

Approximately 40–50% of CRC patients develop metastasis, mostly to the liver and lung. In cancer patients, metastases are associated with 90% of all cancer-related death; thereby, the mechanisms accounting for the metastatic spread have been deeply investigated. Zhang et al. [27] demonstrated that the rhomboid domain containing 1 (*RHBDD1*) plays a crucial role in driving metastasis formation in CRC patients, via the Wnt/β-catenin pathway. It has been shown that *RHBDD1* is able to influence the Wnt/β-catenin cascade by increasing the phosphorylation of β-catenin at the Ser552 and Ser675 residue without affecting its nuclear translocation. Moreover, it promotes EMT, stemness, migration and invasiveness. *RHBDD1* also improves the expression of the β-catenin target gene, *ZEB1*. Furthermore, the protein level of *RHBDD1* positively correlated with *ZEB1*. Thereby, *RHBDD1* has been proposed as a novel therapeutic target and/or a clinically useful biomarker for metastatic CRC (Table 1).

*SLC35C1*, or GDP-fucose transporter 1, is a member of the solute carrier (*SLC*) superfamily of solute carriers. Deng’s group [28] explored the mechanism throughout *SLC35C1* that regulates the canonical Wnt/β-catenin pathway in CRC. They demonstrated a reduction in *SLC35C1* and an increase in β-catenin at all tumour stages. Indeed, silencing *SLC35C1* resulted in the increased release of Wnt3a and *c-myc*, *Axin2* and *cyclin-D1* expression. This suggests that *SLC35C1* is involved in the control of the canonical Wnt/β-catenin pathway, and thereby in tumour cell proliferation and tumour progression (Table 1). 

Neuronal pentraxin 2 (*NPTX2*) is a member of the neuronal pentraxin family and is essential for the formation of synapsis. *NPTX2* was found overexpressed at both mRNA and protein level in CRC, particularly in metastatic lesions [29]. *NPTX2*, which was found to positively correlate with tumour stages, lymphatic invasion, distant metastasis, and poor patients’ outcome, promotes β-catenin nuclear translocation and the expression of *c-myc*, *cyclin D1, Snail*, and *N-cadherin*. No *NPTX2* receptors have been identified in CRC; however, its cellular internalization was found mediated by the Wnt/β-catenin receptor, *FZD6*. Additionally, it has been reported that *NPTX2/FZD6* interaction translates in cancer cell proliferation and metastasis formation by triggering the Wnt/β-catenin pathway [29] (Table 1).

Aberrant gene expression and DNA methylation profiles are considered hallmarks of CRC initiation and progression [77]. Due to the *APC* inactivating mutations, the Wnt/β-catenin pathway plays a key role in CRC metastatic spread [78]. Bruschi el al. [79] investigated the early transcriptional and epigenetic changes resulting from *APC* inactivation in intestinal crypts in crypt base columnar (*CBC*) cells. The authors have found that *APC* disruption rapidly induces changes in DNA methylation, indicating that focal remodelling of the DNA methylation profile occurs early and concomitantly with the first oncogenic event. Moreover, it has been demonstrated that the hyper-activation of the Wnt/β-catenin pathway associated with the *APC* loss-of-function turns out in a rapid increase in intestinal stem cell commitment towards differentiation. Again, it was correlated with the remodelling of the DNA methylation profile. This study unveils that early changes in DNA methylation are crucial for the impaired fate decision program associated with *APC* loss-of-function.

The kelch-like family member 22 (*KLHL22*) is a tumour suppressor protein involved in the development/progression of several cancers [30]. Low expression of *KLHL22* was found in CRC tissues. *KLHL22* overexpression was associated with decreased migration, invasion and reduced expression of the EMT markers, vimentin, N-cadherin, Twist1 and Snail1. Intriguingly, *KLHL22* knockdown led to increased expression of β-catenin and *LEF*, while *KLHL22* overexpression translates into *GSK-3β* upregulation and β-catenin downregulation [30] (Table 1).

## 6. Non-Canonical Wnt Pathway and CRC

The canonical and non-canonical Wnt family members play discrete roles in CRC. The activation of the Wnt/calcium pathway turns into stimulation of sensitive proteins such as *CamKII* and *PKC* [80]. A Ror family of receptor tyrosine kinases, the *ROR2* has been shown to act as a Wnt5a receptor or co-receptor [81]. Wnt5a has different roles in CRC. It can act as an antagonist or agonist of the canonical Wnt/β-catenin pathway, depending on the cellular context. Lee et al. [82] noticed that the antagonism between the canonical and the non-canonical Wnt/β-catenin signalling pathways is linked to Wnt5a. Mechanistically, Wnt5a suppressed the canonical Wnt/β-catenin cascade by acting as a ligand on the *RORα* [81]. After *PKCα*-mediated phosphorylation, RORα modifies its affinity and interacts with the armadillo repeat domains of β-catenin, thus supressing its transcriptional activity.

Three relevant goals have been recently achieved by Voloshanenko et al. [83] supporting the role of Wnt5a/b in cell growth, via the non-canonical β-catenin pathway. First, they identified the procollagen-lysine,2-oxoglutarate 5-dioxygenase 2 (*PLOD2*), the hydroxyacyl-CoA dehydrogenase (*HADH*), ligand-dependent corepressor (*LCOR*) and the receptor expression-enhancing protein 1 (*REEP1*) as candidate genes regulated by the non-canonical Wnt/β-catenin pathway. Second, these genes were found regulated by Wnt5a/b, as well as by *ROR2*, the *DVL2*, the activating transcription factor 2 (*ATF2*) and *ATF4* in a non-canonical Wnt/β-catenin independent manner. Lastly, Wnt5a/b silencing was found to impair cancer cell proliferation.

Among several soluble Wnt proteins, Wnt11 was found to be upregulated in CRC patients [84]. Recently, Gorroño-Etxebarria and colleagues [85] have shown that increased Wnt11, and its *FZD6*, *RYK, PTK7* receptors, positively correlate with poor prognosis. Additionally, Wnt11 downregulated β-catenin transcriptional activity and increased *ATF2* via the non-canonical Wnt signalling pathway. Thereby, Wnt11 has been proposed as a prognostic biomarker and therapeutic target in CRC patients.

Tumour micro environment (TME) has a pivotal role in cancer development [86]. Liu et al. [31] reported that, unlike CRC cells, tumour associate macrophages (TAMs), and, in particular, M2-like cells, express Wnt5a. Furthermore, it has been shown that Wnt5a positive TAMs regulate macrophages infiltration, tumour cell proliferation and migration. Wnt5a pro-tumour activity was found to be associated with the overexpression of the C-C motif chemokine ligand 2 (*CCL2*) in Wnt5a-treated macrophages. Consistently, Wnt5a knockdown reduced *CCL2* expression in TAMs and their cancer-promoting activity. In Wnt5a-treated macrophages, both *CaMKII* and ERK1/2 undergo phosphorylation and lead to *CCL2* secretion. This study provided evidence for a new role of Wnt5a in CRC and describes a potential novel therapeutic target (Table 1).

## 7. Breast Cancer

Breast cancer (BC) is the most diagnosed cancer in women [87], the first cause of cancer death in women worldwide [88], and one of the most expensive in terms of health care costs [87]. Both the canonical and non-canonical Wnt/β-catenin pathways are essential for mammary gland development [89] and for BC growth and dissemination [90]. Hyper-active Wnt/β-catenin was reported in breast tumours [91]. In human BC, elevated intracellular β-catenin level has been associated with high tumour grade [92] and poor prognosis. In addition, up to 90% of metaplastic carcinomas and non-metastasizing fibromatosis have been associated with the highest β-catenin expression level [93]. Moreover, proteins such as Wnt3a [94] and xenopus frizzled 7 (*Xfz7*) [95] have been involved in the activation of both the canonical and the non-canonical Wnt signalling pathways. 

## 8. Canonical Wnt Pathway and BC

Dysregulation of the Wnt/β-catenin cascade has been associated with cancer initiation and metastasis formation [96]. Moreover, high β-catenin expression has been reported in basal-like BC subtype [91]. Additionally, it has been demonstrated that loss of secreted frizzled-related protein 1 (*sFRP1*) is an early event in BC patients and is associated with poor prognosis [97]. Furthermore, the activation of the Wnt/β-catenin cascade has been associated with radio resistance of progenitor cells. Thereby, the Wnt/β-catenin pathway has been proposed as a target to harm the self-renewal potential of stem/progenitors [98]. 

A recent study demonstrated that high β-catenin level is associated with miR106a overexpression and involved in BC cell growth. Additionally, high levels of miR106a were reported to reduce cisplatin sensitivity. Major results were obtained exploiting the Wnt inhibitor, FH535. In fact, FH535 treatment reduced the expression of β-catenin, *cyclin D1*, *c-myc* and *Ki67*, impaired tumour growth and induced apoptosis [64].

In a different study [99], the impact of the Wnt/β-catenin canonical pathway in cisplatin resistance was investigated by silencing β-catenin via small interfering RNA (siRNA). The authors demonstrated that upon β-catenin silencing, the cells become more sensitive to cisplatin treatment. These effects were associated with the increased expression of the apoptotic proteins caspase 3/9. 

A recent study demonstrated that miR-5188, aberrantly expressed in breast cancer patients, positively correlates with poor prognosis. The molecular analyses revealed that miR-5188 directly targets the forkhead box protein O1 (*FOXO1*). In the physiological setting, *FOXO1* binds β-catenin and induces its degradation. This implies that miR-5188 overexpression leads to β-catenin nuclear accumulation and transcription of its downstream target genes, mainly involved in EMT, tumour cell proliferation, metastasis formation and chemo resistance. Moreover, the authors elegantly showed that miR-5188 expression is under the control of c-Jun, which directly binds to its promoter region. This in turn generates a positive loop, accelerating tumour progression. Clinically, miR-5188 has been proposed as a diagnostic or prognostic factor and/or a direct target for anti-cancer therapy [65].

The upregulation of the lncRNA hoxa transcript at the distal tip (*HOTTIP*) has also been linked to poor prognosis in BC patients. Overexpression of *HOTTIP* correlates with the expansion of breast CSCs (BCSCs) and the expression of the stem cell markers, *OCT4* and *SOX2*. Han et al. [66] demonstrated a reduced expression of differentiation markers, such as *CK18* and *CK14* and that miR-148a inhibits BC cell migration and invasion by directly targeting Wnt1. Moreover, it has been reported that *HOTTIP* controls miR-148a-3p by acting as a competing endogenous RNA (ceRNA). Thereby, *HOTTIP* promotes expansion of CSCs in vitro and tumorigenesis in vivo by regulating the miR-148a-3p/Wnt1/β-catenin axis [66]. These data are summarized in Table 2.

The *LGR4* was identified as a prognostic marker in breast tumours displaying poor prognosis [32]. A tight molecular interplay between *LGR4* and Wnt/β-catenin signalling has been reported to control stemness. Indeed, *LGR4* binding to the soluble R-spondin proteins eases the Wnt/β-catenin cascade [33]. Previous studies have proven that upregulation of *ZEB1* by *SLUG* (the protein product of *SNAI2*), increased EMT [26]. As a matter of fact, *LGR4* knockdown leads to *SLUG* and *ZEB1* downregulation, thereby impairing invasion and metastasis [17]. A correlation with poor outcome and the expression of the *LGR4* homolog *LGR5* was also reported. *LGR5* maintains the pool of BCSCs and promotes tumour progression and invasiveness by activating the Wnt/β-catenin canonical pathway [17] (Table 1).

Wang et al. [34] first demonstrated that the expression of the suppression of tumorigenicity 7 like (*ST7L*) is downregulated in BC cells, and more importantly, that *ST7L* acts as an antitumor supervisor by reducing *GSK-3β* phosphorylation and inducing β-catenin degradation. However, the mechanisms through which *ST7L* controls *GSK-3β* phosphorylation are still missing (Table 1).

A recent study [35] reported the overexpression of the transmembrane emp24 domain (*TMED*) in BC and its correlation to poor prognosis. An aberrant level of *TMED* boosts cell cycle progression, colony formation, migration and invasion and the expression of *CDK2*, *CDK4*, *CDK6*, cyclin E, β-catenin, *cyclin D1*, *c-myc*, *MMP-7* and *TCF4*. Conversely, silencing *TMED3* drastically reduced migration and invasion. Moreover, the observation that β-catenin knockdown translates in the reduction of its regulated genes supports the notion that the oncogenic effect of *TMED* goes through the Wnt/β-catenin pathway (Table 1).

Cryptotanshinone (CTS) is an herbal medicine derived from roots of salvia miltiorrhiza, which displays anti-tumour properties. It has been shown that in vitro CTS reduces tumour cell growth, migration and invasion by downregulating the pyruvate kinase muscle isozyme M2 (*PKM2*), a protein involved in glycolysis, and more importantly in β-catenin activation [100].

## 9. Wnt Non-Canonical Pathway and BC

Among the Wnt ligands, the most extensively studied ligand, activating the β-catenin independent pathway, is Wnt5a. However, its different biological actions are enlightened by the observation that it can also initiate the canonical β-catenin signalling cascade [101].

Wnt5a is an evolutionarily conserved Wnt ligand, which plays an important role in developmental processes. Wnt5a^-/-^ knockout mice showed perinatal lethality, due to developmental defects [102].

In tumorigenesis, Wnt5a signalling is central and displays multiple intriguing and opposite roles, mainly acting as a β-catenin antagonist. These data are discussed.

The Wnt5a suppressive properties detected in tumours connoted by β-catenin hyper-activation have been linked to the shift towards the stimulation of the β-catenin independent signalling pathway.

Foxy5 is a Wnt5a mimicking hexapeptide able to decrease BC cell migration and invasion [103]. More recently, Prasad et al. [36] confirmed these data and added new information on the role of Wnt5a in the regulation of the expression of the phosphofructokinase platelet-type (*PFKP*). They have shown that low *PFKP* level correlates to cancer cell migration and poor patients’ survival. The growth and expansion of tumour cells also rely on glucose consumption, resulting in the accumulation of lactate. Cancer cell metabolism was also associated with β-catenin activation [37]. In this regard, it has been shown that Wnt5a affects the aerobic glycolysis by inhibiting the activation of β-catenin. Therefore, an onco-suppressive role was proposed for *PFKP*.

According to the study of Borcherding et al. [104], Roarty et al. [105] demonstrated that the paracrine activity of Wnt5a suppresses the expression of both β-catenin and *cyclin D1*. The authors have shown that Wnt5a supports *TGF-β*-mediated tumour suppressive functions by antagonising Wnt/β-catenin signalling and limiting tumour cell proliferation.

Moreover, Leris and colleagues [38] proved that Wnt5a mRNA level was significantly lower in tumour than in normal tissues, particularly in those displaying a more aggressive behaviour. Again, this observation has suggested a suppressive role of Wnt5a in cancers. It has also been reported that loss of Wnt5a is associated with a higher histological tumour grade, increased risk of recurrence, and a shorter recurrence-free survival in invasive BC [39] (Table 1).

On the contrary, Kobayashi et al. [40] reported that Wnt5a is expressed in ER-positive BC cells and positively associates to vessel invasion, tumour size and migration. Mechanistically, Wnt5a induces the expression of the activated leukocyte cell adhesion molecule (*ALCAM*), a protein involved in migration and invasion. Knockdown of either Wnt5a or *ALCAM* inhibited tumour cell migration, confirming the role of the Wnt5a/*ALCAM* axis in the migratory phenotype of ER-positive BC (Table 1).

A relevant role of Wnt5a in reprogramming the TME was also described [106]. It has been shown that under pro-inflammatory conditions the non-canonical Wnt protein induces the expansion of the CD163(+) immunosuppressive macrophages translating in the release of IL-10 and the inhibition of the classical *TLR4*-NF-kB signalling pathway [106].

Moreover, higher level of Wnt5a was found in human monocyte-derived myeloid dendritic cells (Mo-mDCs) than in normal monocytes and macrophages. Wnt5a was found to inhibit the generation of Mo-mDCs by stimulating BC cells to produce IL-6. In addition, the presence of IL-6 in the conditioned media of Wnt5a stimulated BC cells was found to be involved in the inhibition of Mo-mDC differentiation [107]. Consistently, overexpression of Wnt5a mRNA was detected in metastases derived from primary BC cells and in BC cell lines [108].

Wnt5a signalling is also able to modify the CD44-AKT signalling pathway, leading to a reduced BC cell migration and invasion. In epithelial BC cells, silencing of Wnt5a drives EMT-like changes without altering the expression of common EMT markers. On the contrary, it interferes with CD44 expression and induces pAKT downregulation, thereby acting via a EMT-independent mechanism [109]. 

The dual activity of Wnt5a has also been ascribed to the Wnt5a isoforms. Bauer et al. [110] have shown that the Wnt5a gene encodes for two distinct isoforms: the Wnt5a-long (*Wnt5a-L*) and Wnt5a-short (*Wnt5a-S*) isoform. When analysed in several cell lines, *Wnt5a-L* reduced tumour progression, while *Wnt5a-S* promoted tumour growth. 

Overall, Wnt5a may play multiple roles. Whether it acts as a tumour suppressor or a tumour promoter remains elusive and depends on the availability of essential receptors, the TME, and the activation of discrete signalling pathways.

## 10. Triple-Negative Breast Cancer

Triple-Negative Breast Cancer (TNBC) is an invasive type of breast carcinoma that lacks the expression of estrogen and progesteron receptor as well of the human epidermal growth factor receptor 2 (HER2) [111] and accounts from 10 to 15% of all BC [112].

TNBC patients have poor outcome due to the high grade of proliferation, early tumour dissemination, and the lack of targeting approaches [113,114]. The malignancy is associated with earlier age of onset, aggressive clinical course, and dismal prognosis [112]. TNBC gained attention due to the aggressiveness and the lack of effective treatment options. Therefore, the most relevant data on this breast cancer subtype are independently discussed.

Gene expression omnibus (GEO) databases were applied by Shen et al. [41] to gather gene expression data in TNBC patients who underwent chemotherapy. They reported that co-expression of NIMA-related kinase 2 (*Nek2*) and β-catenin correlated with patients’ poor prognosis. β-catenin binds to and is phosphorylates by the *Nek2B* isomer. Thereby, in TNBC, *Nek2B* functions as a β-catenin regulator by activating the Wnt signalling pathway and its downstream target genes. In addition, it has been suggested that *Nek2B* and β-catenin may synergize to promote resistance to chemotherapy. However, further studies are required to better elucidate the relationship between β-catenin and *Nek2* and its possible implications in cancer development (Table 1).

TNBC aggressiveness also relies on the activation of the non-canonical Wnt/PCP pathway. Indeed, the aberrant activation of downstream genes activated by the non-canonical Wnt/PCP pathway has been implicated in tumour growth and poor prognosis. Results from Puvirajesinghe and colleagues [42] revealed that Van Gogh-like 2 (*VANGL2*), a core Wnt/PCP component, plays a crucial role in cancer cell migration, anchorage-dependent and independent cell proliferation, as well as in tumour growth. Since the scaffold p62/SQSTM1 protein, a *VANGL2*-binding partner, has a key role in the *VANGL2*–p62/SQSTM1–*JNK* pathway, the possibility to exploit p62/SQSTM1 as a potential therapeutic target has been proposed. This would be of particular relevance since the *JNK* targeting approaches are associated with major side effects in the clinical setting (Table 1).

Yu and colleagues [43] demonstrated that the hematopoietic protein tyrosine phosphatase (*HePTP*) stabilizes β-catenin in the cytoplasm and allows its nuclear translocation by regulating the phosphorylation of *GSK-3β*. This results in the transcriptional activation of target genes, leading to cell migration and invasion. Since knockdown of *HePTP* significantly suppresses metastases formed by TNBC cells, *HePTP* has also been proposed for therapeutic approaches in TNBC (Table 1).

Recently, Kong et al. [44] have shown that a Rho-GTPase-activating protein, the deleted in liver cancer gene 3 (*DLC-3*), is downregulated in TNBC and its expression is linked to lymphatic metastases. *DLC-3* overexpression leads to β-catenin and *c-myc* downregulation as well as in reduced in vitro cell proliferation, colony formation, migration, and invasion. Hence, a tumour-suppressor role related to the inhibition of the Wnt/β-catenin signalling pathway has been postulated (Table 1).

Liu and colleagues [67] have reported a low expression of miR-6838-5p in TNBC compared to normal cells. miR-6838-5p overexpression reduced cell invasion, migration, EMT, β-catenin, *c-myc* and *cyclin D1* expression by post-transcriptionally controlling Wnt3a expression.

Recently, miR-27a-3p was found overexpressed in tumour cells and linked to poor prognosis in TNBC patients. miR-27a-3p leads to the activation of Wnt/β-catenin cascade and enhances cell proliferation and migration by directly targeting the 3ʹ-UTR region of *GSK-3β* [68] (Table 2). 

## 11. Ovarian Cancer 

Ovarian Cancer (OC) is a global issue representing the fourth most common cancer in the female population, particularly in developed countries [115]. The poor survival rate is mainly due to the lack of screening methods at the early stages along with the absence of effective treatment options for advanced stages [116]. Among different OC subtypes, the epithelial subtype (EOC) holds about 90% of the overall ovarian malignancies [117]. 

## 12. Canonical Wnt Pathway and OC

The Wnt/β-catenin signalling pathways play a crucial role in carcinogenesis of all OC subtypes [118]. In particular, several transcription factors, proteins and miRNAs acting on this pathway have been explored [119].

Chen and co-workers [45] investigated the role of the Wnt/β-catenin pathway antagonist dickkopf-related protein 1 (*DKK1*). They showed that *DKK1* is involved in the control of OC stemness. Mechanistically, it has been shown that STAT3 directly activates the transcription of miRNA-92a, translating in *DKK1* downregulation [45]. Moreover, overexpression of miR-1207 was found to correlate with high nuclear β-catenin level [46]. Wu et al. [46] investigated the effects of miR-1207 on the expression of the *SFRP1*-*AXIN2* and the inhibitor of β-catenin and T cell factor 4 (*ICAT*). They found that miR-1207 overexpression was associated with a reduced *SFRP1-AXIN2* and *ICAT* expression and the appearance of a stem-like phenotype (Table 1). 

Salem et al. [69] proved that miR-590-3p promotes OC growth and metastasis, by targeting *FOXA2*. Moreover, it has been shown that miR-590-3p upregulation significantly increases cell growth, migration, and invasion in EOC cells, both in vitro and in vivo [70]. Similarly, *FOXA2*, which exhibits suppressive activity on EOC cells, has been identified as a miR-590-3p target [70]. The cyclin G2 gene (*CCNG2*) has also been reported to display several repressive actions on EOC-derived tumour cell lines. It inhibits cell proliferation, migration, invasion and EMT. Thereby, since miR-590-3p post-transcriptionally regulates *FOXA2*, *FOXO3*, *CCNG2* and *DDK1* expression, miR-590-3p has been proposed as a potential target in EOC patients [70]. A crucial role of *SFRP1* in OC growth has also been proposed. Since miR-1180 is highly expressed in neoplastic tissues, Hu et al. [71] explored the relationship between miR-1180 and the *SFRP1*/Wnt/β-catenin signalling pathway in this context, demonstrating that miR-1180 triggers the activation of the Wnt/β-catenin cascade by targeting *SFRP1*. 

The members of the R-spondin ligand family have been reported as positive effectors of Wnt/β-catenin signalling [47]. *LGR4-6* plays crucial roles in the activation of the Wnt/β-catenin cascade [47,48]. Moreover, Ruan et al. [47] have reported that LGR6 induces stemness and chemo resistance via the Wnt/β-catenin pathway in OC cells. Restrain of the stem phenotype and increased sensitivity to chemotherapy have been proved by *LGR6* silencing (Table 1).

A recent study established that the overexpression of the Rab GTPase family member, *Rab14*, regulates *GSK-3β* phosphorylation and β-catenin nuclear accumulation [49,50]. Moreover, high levels of *Rab14* were found to be associated with higher expression of Wnt/β-catenin target genes including *MMP-7* and *c-myc* [50] (Table 1).

Jiang et al. [115] have demonstrated that tetrandrine (TET) enhances the anti-tumour effect of paclitaxel (PTX) by decreasing *c-myc* and *cyclin D1* and increasing p21 expression, resulting in cell cycle arrest. The pro-apoptotic effects of PTX+TET have also been investigated. TET was found to inhibit β-catenin downstream target genes by enhancing PTX activity and conferring sensitivity to PTX in resistant cells [115]. 

Barghout and co-workers [120] demonstrated a more active Wnt/β-catenin signalling in carboplatin-resistant cells than in sensitive ones. Unlike the Wnt ligands, the negative Wnt regulators *DKK1*, *SFRP1*, and the *FRZB* have been found downregulated in cisplatin-resistant cells. These findings suggest that Wnt/β-catenin blockade may be effective on resistant EOC.

## 13. Non-Canonical Wnt Pathway and OC

*FZD7* is highly expressed in OC [51], and its overexpression in mesenchymal (Mes) and Stem-A OC subtypes has been associated with the induction of EMT. The PCP pathway, which activates the *Rho–ROCK* axis, was found to be involved in the activation of actomyosin contractility, cadherin-based cell-cell adhesion and migration, while the Wnt/calcium pathway in the metastatic spread and cytoskeleton changes in this clinical setting [51]. Therefore, it has been proposed that the *FZD7* controls both cell cycle progression and cell migration via the non-canonical Wnt/PCP pathway (Table 1).

The integrin beta like 1 subunit (*ITGBL1*) was found to be highly overexpressed in OC [52]. It has been shown that *ITGBL1* promotes cell migration and adhesion via Wnt/PCP, *RhoA*, the focal adhesion kinase, and the steroid receptor coactivator (*FAK/src*) pathway (Table 1).

The *PTK7*, which interacts with Wnt5A, *LRP6* and *FZD7* [121,122], may act as a tumour suppressor or oncogene [123,124]. In EOC, *PTK7* downregulation is indeed associated with a poor prognosis [123].

Luo and colleagues [53] have investigated the role of the alkaline phosphatase (*ALPL*) in OC. They demonstrated that *ALPL* overexpression inhibits EMT, migration and invasion of high grade serous OCs (HGSOC) and *FZD2* correlates with a poor survival rate [53]. Mechanistically, they have shown that ALPL overexpression represses Wnt5a/*FZD2*–mediated EMT activation, possibly by interfering with STAT3 activation [53] (Table 1).

## 14. Wnt Pathway and Other Cancers

Glioma is an aggressive tumour of the nervous system displaying rapid progression and poor prognosis. Zhao et al. [125] have found that overexpression of β-catenin and *cyclin D1* is associated with high level of the long noncoding RNA, *FGD5* antisense RNA 1 (lncRNA FGD5-AS1). A close relationship between them was straitened by the observations that inhibition of FGD5-AS1 reduced β-catenin and *cyclin D1* expression while β-catenin downregulation decrease lncRNA FGD5-AS1 expression. This results in the impaired tumour cell migration and invasion.

Prostate cancer (PCa) is among the most common tumour in male. A recent study by Situ et al. [72] provided evidence for the involvement of the microRNA-939 (miR-939) in PCa. Downregulation of miR-939 was found in tumour tissues at advanced tumour stage, in distant lesions, as well as being associated with poor prognosis. Molecularly, it was demonstrated that miR-939 upregulation interferes with the Wnt/β-catenin cascade by directly targeting the hepatoma-derived growth factor (*HDGF*).

Osteosarcoma (OS) is a common bone paediatric tumour displaying high rates of lung metastasis. The inhibition of β-catenin activation, metastasis formation and chemo-resistance were found modulated by tegavivint (a Wnt/β-catenin inhibitor), which has been proposed as an alternative therapeutic option in OS [126].

Melanoma is among the most immunogenic tumours displaying increased lymphocytic infiltration. Low 1α,25-dihydroxyvitamin D3 and vitamin D receptor (*VDR*) level correlates to increased cancer incidence and melanoma progression, respectively. Recently, it has been shown that high *VDR* expression correlated with the inhibition of tumour growth, low Wnt/β-catenin activation and the induction of the immune response [54] (Table 1).

The long non-coding RNA00261 (Linc00261) has been shown to display onco-suppressor properties in Pancreatic Cancer (PC). Linc00261 overexpression inhibits PC cell proliferation, invasion, EMT and metastasis. Bioinformatics analysis revealed that Linc0026 inhibits the activation of the β-catenin/*TCF4* cascade and the metastatic spread by regulating the miR-552 5p/*FOXO3* axis [127].

## 15. Extracellular Vesicles and the Wnt Pathway 

EVs are heterogeneous small membrane-bound carriers with complex cargoes released under both physiological and pathological conditions. Almost any cell can release EVs, which act as inter-cellular mediators modifying target cell fate at closed or distant sites [128].

Based on the biogenesis, size, content, mechanisms of release and function, three discrete EV subtypes are recognized: microvesicles (MVs), exosomes, and apoptotic bodies [128]. 

**Figure 3:**
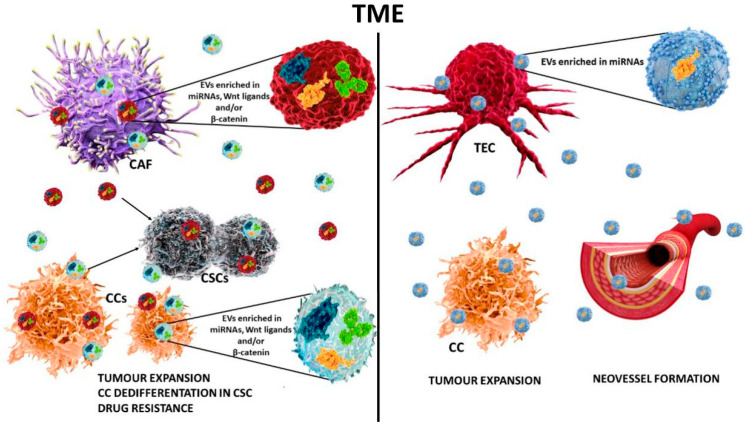
Schematic representation of cell-to-cell communication in the TME by EVs. EVs are released by almost all cell types in the TME. EVs serve as inter-cellular mediators transferring specific molecules (proteins including Wnt ligands and β-catenin, and miRNAs) to recipient cells, thus promoting tumour expansion, cancer cell dedifferentiation in CSCs, chemo-resistance, and neovessel formation. CCs: cancer cells; CSCs: cancer stem cells; TEC: tumour-derived endothelial cell; CAF: cancer associated fibroblasts.

EVs-mediated transfers of specific molecules are known to dictate the phenotype of the recipient cell. They can act on proliferation, motility, EMT, migration, invasion, immune evasion, chemo-resistance, and TME reprogramming (Figure 3).

Moreover, EVs derived from serum or other biological fluids have been proposed as tumour biomarkers. More importantly, EVs have gained attention as anti-cancer tools. Indeed, EVs can be used as drug delivery systems or potential cancer vaccines. Moreover, the transfer of Wnt ligands or β-catenin via EVs has been proposed as a Wnt signalling activation mechanism.

Kalra et al. [129] have shown that EVs released by CRC cells and containing the mutant β-catenin and high Wnt/β-catenin activity boost the expression of target genes as *c-myc* and *cyclin D1* when transferred to recipient cells (Table 3). 

The *14-3-3* are conserved molecules displaying regulatory functions and promoting cancer progression [130]. The *14-3-3ζ* isoform, which binds both β-catenin and *GSK-3β*, leads to the nuclear translocation and accumulation of β-catenin and enhances cell motility. Moreover, EVs enriched in *14-3-3ζ* and β-catenin, after internalization, promote cell survival and migration by activating the Wnt/β-catenin cascade [130] (Table 3).

Hu et al. [131] have investigated the mechanism of drug resistance in CRC and have proven that EVs released by fibroblasts drive dedifferentiation of CRC cells towards CSCs (Figure 3a). Additionally, they found that EVs derived from fibroblasts contain the Wnt ligands that activate the Wnt/β-catenin pathway in target cells, induce transdifferentiation of CRC cells into CSCs and increase drug resistance. Furthermore, it has been reported that collagen accumulation and the *APC* mutation in CRC cells stimulate the release of EVs and, under hypoxia conditions, fibroblast derived EVs boost CRC colony formation [132] (Table 3).

Accumulating evidence shows that EVs enriched in miRNAs are key determinants of human cancer cell growth, invasion and metastasis [73]. CAF-derived EVs enclose miR-92a-3p, which contributes to cancer progression, stemness, EMT, and drug resistance. Moreover, miR-92a-3p enriched EVs correlated with the activation of the Wnt/β-catenin pathway [73] (Figure 3a).

Long non-coding RNA-*APC1* (lncRNA-APC1) is a negative regulator of CRC. Low levels of lncRNA-APC1 correlate with metastasis, advanced clinical stage and poor prognosis in CRC patients. *APC*, via lncRNA-APC1, promotes cell-cycle arrest and suppresses angiogenesis by lowering the release of CRC cell-derived EVs. Finally, it has been shown that EV-derived from CRC are enriched in Wnt1 and enhance CRC cell proliferation and migration via non-canonical Wnt/PCP signalling [139].

Hepatocellular carcinoma (HCC) is one of the most common causes of cancer-related deaths worldwide. Constitutive activation of the Wnt/β-catenin pathway turns into the expression of the epithelial cell adhesion molecule (*EpCAM*) [133]. Ishiguro et al. [134] provided evidence that loss in β-catenin and reduced proliferation and invasion can be obtained by *EpCAM* positive liver cancer stem cells (LCSC) targeted by EVs engineered with a β-catenin specific siRNA (Table 3).

Multiple myeloma (MM) is a hematopoietic malignancy associated with an altered homeostasis of bone formation/resorption. MM-derived EVs enriched in *DKK-1* were found to boost the Wnt/β-catenin signalling and contribute to the abnormal osteogenesis. The inhibition of EV shedding, combined to chemotherapy, was found to impair tumour load, angiogenesis and osteolysis [135] (Table 3).

Furthermore, a recent study noticed that the release of EVs from HCC cells is increased in hypoxic conditions and linked to cancer cell proliferation, migration, invasiveness and EMT. Mechanistically, they have shown that miR-1273f enriched in EVs activates the Wnt/β-catenin signalling cascade by targeting the Wnt/β-catenin inhibitor LHX6 [74].

Chen et al. [136] proved that EVs released from oral squamous cell carcinoma (OSCC) cells correlate with an increased level of β-catenin, the expression of several oncogenic markers, the reprogramming of normal gingival fibroblasts into CAFs, increased metastasis, stemness reprogramming, chemoresistance, and poor patients’ survival (Table 3). 

Xia et al. [75] have demonstrated the uptake of EVs and the delivery of functional miRNAs in different cell lines. The exosomal-miR-1260b was found to be crucial for the activation of the Wnt/β-catenin signalling and the invasivness of lung adenocarcinoma cells.

Harada et al. [137] purified and characterized Wnt5b-associated EVs. In pancreatic PANC-1 and colorectal Caco-2 cell lines, Wnt5a carried by EVs displays the ability to enhance cancer progression (Table 3).

Luga et al. [138] demonstrated that EV shedding by fibroblasts boosts BC cell growth and motility via Wnt/PCP signalling. CAF-derived EVs were found to be crucial drivers of cell migration during metastasis formation. Moreover, they found that EVs secreted from fibroblast L cells promote the autocrine Wnt11-PCP cascade in tumour cells, increasing their motility and metastatic properties (Table 3).

Lombardo et al. [76] provided evidence that EVs released by tumour-derived endothelial cells (TECs-EVs) boost in vivo TEC-derived neovessels. Mechanistically, they showed that EVs released by naive TECs-EVs regulate the expression of *APC*, *GSK-3β* and drive β-catenin nuclear accumulation via miR-214-3p and miR-24-3p (Figure 3b). Overall, this study revealed a key role of the Wnt/β-catenin cascade in TEC-derived neovessel formation. Moreover, they recently showed that naïve TEC-EVs were also able to boost TNBC metastatic spread and lung metastasis formation when injected intravenously [140] (Table 2).

Overall, these data indicate a crucial contribution of EVs released by different cell sources in driving tumor development and dissemination. Several data suggest that these effects mainly rely on the transfer of their specific cargo into target cells. Therefore, approaches able to modify their cargo, particularly miRs and proteins involved in their tumor promoting action, have been proposed as useful therapeutic options. EV engineering by using siRNA for mutated protein has been tested and its effectiveness demonstrated in pancreatic cancer [141]. This suggests that using siRNA for mutant β catenin should be considered as an alternative option for CRC. Likewise, siRNA for different Wnt proteins or rearrangement of dysregulated EV miRs can be used to target the Wnt/β catenin cascade. Alternatively, EVs loaded with Wnt/β catenin inhibitors can be used as natural delivery tools.

## 16. Conclusions

Cell-to cell communication is part of the evolutional processes. Wnt ligands are essential for homeostasis and, in the last 30 years, genetic, biochemical, and molecular investigations have uncovered several Wnt signalling components [2,3]. Driving interest on this topic mainly relies on dysregulation of the Wnt/β-catenin signalling and cancer development/progression [3]. Moreover, Wnt/β-catenin cascade seems to contribute to the TME shape, which plays a crucial role in the control of tumour progression and immune regulation. Many different Wnt proteins have been described, and, among them, Wnt5a plays a critical role, taking part in both the canonical and the non-canonical Wnt/β-catenin pathway [104,105].

The identification of specific tools able to interfere with the Wnt/β-catenin cascade has been a hotspot for many years. This is particularly true for CRC, in which almost 70% of CRC patients display *APC* mutations [15]. Apart from CRC, the Wnt/β-catenin pathway is gaining attention in several malignancies, such as breast, ovarian, melanoma, prostate and paediatric osteosarcoma [53,124,125]. In this regard, BC and in particular TNBC are featured by the abnormal activation of both the canonical and non-canonical Wnt/β-catenin pathway [113,114]. Likewise, a hyper-active Wnt/β-catenin cascade has been shown to play a crucial role in the progression, stemness, and drug resistance in OC [70,119]. Several miRNAs have been identified to modulate this cascade and thereby widely studied as screening markers or targets in different tumour settings [142].

In the TME, intercellular communication has been recently reported as mediated by the transfer of EV molecular cargo and revised in [143]. Their cargo also includes a number of Wnt components. Of note, wild-type and mutant β-catenin, able to promote survival and proliferation of recipient cells and, in several instances, dedifferentiation towards a CSC phenotype, have been detected in EVs (Figure 3a). Moreover, their role in mediating drug resistance has been reported. Furthermore, since EVs are released within the TME, their contribution in cancer growth and progression has been extensively investigated [144]. EV shedding, blockade, or engineering have been proposed as innovative anti-tumour instruments for fine-tuning the Wnt/β catenin pathway [142,145]. 

In recent decades, several efforts have been directed to the development of Wnt/β catenin targeting approaches in order to interfere with tumour progression. However, these efforts have been limited by the crucial role of the Wnt/β catenin pathway in preserving tissue homeostasis. Therefore, future energies should be directed to clearly dissect the mechanisms driving the unbalanced Wnt/β catenin pathway in cancer, and the EV mechanism of action should be considered amid them. Should they be identified, targeting approaches would become a suitable anti-cancer option. 

## Figures and Tables

**Table 1:** Proteins involved in several tumours, their alteration, targets, and impact on tumours.

Protein	Related Cancers	Expression Level	Pathway Interaction	Impact on Tumour	Ref.
RNF6	CRC	Upregulated	β-catenin	cell growth cell cycle progression EMT metastasis	[15]
LGR5	CRC, BC	Upregulated	β-catenin	proliferation migration	[16,17]
TNIK	Gastric	Upregulated	β-catenin	cell growth	[18,19]
KYA1797K	CRC	Upregulated	β-catenin	tumour growth stem cell features	[20]
BCL6	CRC	Upregulated	β-catenin	cellular proliferation tumour development tumour progression	[21,22,23]
ZEB2 and ZEB1	CRC	Upregulated	β-catenin	tumour progression invasion	[17,24,25,26]
XIAP	CRC	Upregulated	β-catenin	proliferation chemoresistance	[24,25]
RHBDD1	CRC	Upregulated	β-catenin	metastasis stemness EMT migration invasiveness	[27]
SLC35C1	CRC	Downregulated	β-catenin	cell proliferation cell progression	[28]
NPTX2	CRC	Upregulated	β-catenin	tumour stages lymphatic invasion metastasis	[29]
KLHL22	CRC	Downregulated	β-catenin	invasion migration	[30]
CCL2	CRC	Upregulated	Non-canonical	progression	[31]
LGR4	BC	Upregulated	β-catenin	tumorigenesis metastasis CSC maintenance	[17,26,32,33]
ST7L	BC	Downregulated	GSK-3β	proliferation invasion	[34]
TMED	BC	Upregulated	β-catenin	cell cycle progression colony formation migration	[35]
Wnt5a	BC	Downregulated	β-catenin	migration lactate production invasion	[36,37]
Wnt5a	BC	Downregulated	β-catenin -cyclin D1 -TGF-β	cell proliferation aggressiveness	[38,39]
Wnt5a	BC	Upregulated	ALCAM	vessel invasion tumour size migration	[40]
Nek2B	TNBC	Upregulated	β-catenin	chemoresistance	[41]
VANGL2	TNBC	Upregulated	p62/SQSTM1 (PCP)	migration anchorage-dependent and independent cell proliferation	[42]
HePTP	TNBC	Upregulated	-GSK-3β β-catenin	metastasis	[43]
DLC-3	TNBC	Downregulated	β-catenin	proliferation colony formation migration invasion	[44]
DKK1	OC	Downregulated	β-catenin	stemness	[45]
SFRP1	OC	Downregulated	β-catenin	cell growth stem-like phenotype	[46]
AXIN2	OC	Downregulated	β-catenin	stem-like phenotype	[46]
LGR6	OC	Upregulated	β-catenin	stemness chemoresistance	[47,48]
RAB14	OC	Upregulated	β-catenin	proliferation chemoresistance invasion	[49,50]
FZD7	OC	Upregulated	Non-canonical	EMT cell cycle progression migration	[51]
ITGBL1	OC	Upregulated	Non-canonical	migration adhesion	[52]
ALPL	OC	Upregulated	Non-canonical	EMT migration invasion	[53]
VDR	Melanoma	Upregulated	β-catenin	tumour growth immune response	[54]

**Table 2:** miRNAs involved in the tumours, their alteration and tumour impact.

miRNA	Related Cancer	Expression Level	Impact on Tumour	Ref.
miR-144-3p	CRC	Downregulated	cell proliferation	[21,22,23]
miR-377-3p	CRC	Upregulated	cell expansion EMT repression of apoptosis	[60]
miR-377-3p	CRC	Downregulated	proliferation migration chemoresistance	[61]
miR-520e	CRC	Downregulated	cell proliferation colony formation invasion	[62,63]
miR106a	BC	Upregulated	cell growth cisplatin sensitivity	[64]
miR-5188	BC	Upregulated	tumour cell proliferation metastasis formation EMT chemoresistance	[65]
miR-148a	BC	Downregulated	cell migration invasion	[66]
miR-6838-5p	BC	Downregulated	cell invasion migration EMT	[42,67]
miR-27a-3p	BC	Upregulated	proliferation migration.	[68]
miR-1207	OC	Upregulated	tumorigenicity stem cell-like traits stemness	[46]
miR-590-3p	OC	Upregulated	cell growth migration, invasion	[69,70]
miR-1180	OC	Upregulated	cell proliferation glycolysis	[71]
miR-939	PCa	Downregulated	tumour stage metastasis	[72]
miR-92a-3p	CRC EVs	Upregulated	cancer progression stemness EMT drug resistance	[73]
miR-1273f	HCC EVs	Upregulation	cell proliferation migration invasiveness EMT	[74]
miR-1260b	LAC EVs	Upregulation	cell invasion metastasis	[75]
miR-214-3p	TEC EVs	Upregulation	neovessel formation	[76]
miR-24-3p	TEC EVs	Downregulation	neovessel formation	[76]

**Table 3:** EVs involved in several tumours, their alteration, targets, and impact on tumours.

EV Cargo	EV Source	Target Cells	Related Cancers	Expression Level	Pathway Interaction	Impact on Tumour Cells	Ref.
Mutant β-catenin in EVs	LIM1215	RKO	CRC	Upregulated	β-catenin	migration, metastasis tumour growth	[129]
14-3-3ζ in EVs	HEK293T	COS-7, SW480	CRC	Upregulated	β-catenin GSK-3β DVL2	survival migration	[130]
Wnt ligands in EVs	CAFs	CRC	CRC	Upregulated	β-catenin	dedifferentiation drug resistance colony formation	[131,132]
β-catenin in EVs	milk	HCC	HCC	Silenced	β-catenin	proliferation tumour growth	[133,134]
DKK-1 in EVs	MM	MM	MM	Upregulated	β-catenin	osteoclast activity osteoblast differentiation	[135]
EVs	OSCC	OSCC	OSCC	Upregulated	β-catenin	metastasis stemness chemoresistance	[136]
Wnt5b in EVs	Caco-2 and PANC-1	A549	Lung cancer	Upregulated	β-catenin dependent and independent pathways	proliferation migration	[137]
EVs	CAFs	BC	BC	Upregulated	Wnt-PCP	cell growth and motility	[138]
